# The Tumor Immune Landscape and Architecture of Tertiary Lymphoid Structures in Urothelial Cancer

**DOI:** 10.3389/fimmu.2021.793964

**Published:** 2021-12-20

**Authors:** Nick van Dijk, Alberto Gil-Jimenez, Karina Silina, Maurits L. van Montfoort, Sarah Einerhand, Lars Jonkman, Charlotte S. Voskuilen, Dennis Peters, Joyce Sanders, Yoni Lubeck, Annegien Broeks, Erik Hooijberg, Daniel J. Vis, Maries van den Broek, Lodewyk F. A. Wessels, Bas W. G. van Rhijn, Michiel S. van der Heijden

**Affiliations:** ^1^ Department of Medical Oncology, The Netherlands Cancer Institute, Amsterdam, Netherlands; ^2^ Department of Molecular Carcinogenesis, The Netherlands Cancer Institute, Amsterdam, Netherlands; ^3^ Oncode Institute, Utrecht, Netherlands; ^4^ Institute of Experimental Immunology, University of Zurich, Zurich, Switzerland; ^5^ Department of Pathology, The Netherlands Cancer Institute, Amsterdam, Netherlands; ^6^ Department of Medical Oncology, Maastricht University Medical Center, Maastricht, Netherlands; ^7^ Core Facility Molecular Pathology & Biobanking, The Netherlands Cancer Institute, Amsterdam, Netherlands; ^8^ Department of Electrical Engineering, Mathematics and Computer Science, Delft University of Technology, Delft, Netherlands; ^9^ Department of Urology, The Netherlands Cancer Institute, Amsterdam, Netherlands; ^10^ Department of Urology, Caritas St. Josef Medical Center, University of Regensburg, Regensburg, Germany

**Keywords:** immunotherapy, tertiary lymphoid structures (TLS), multiplex immunofluorescence, urothelial cancer, tumor microenvironment, bladder cancer

## Abstract

Candidate immune biomarkers have been proposed for predicting response to immunotherapy in urothelial cancer (UC). Yet, these biomarkers are imperfect and lack predictive power. A comprehensive overview of the tumor immune contexture, including Tertiary Lymphoid structures (TLS), is needed to better understand the immunotherapy response in UC. We analyzed tumor sections by quantitative multiplex immunofluorescence to characterize immune cell subsets in various tumor compartments in tumors without pretreatment and tumors exposed to preoperative anti-PD1/CTLA-4 checkpoint inhibitors (NABUCCO trial). Pronounced immune cell presence was found in UC invasive margins compared to tumor and stroma regions. CD8^+^PD1^+^ T-cells were present in UC, particularly following immunotherapy. The cellular composition of TLS was assessed by multiplex immunofluorescence (CD3, CD8, FoxP3, CD68, CD20, PanCK, DAPI) to explore specific TLS clusters based on varying immune subset densities. Using a k-means clustering algorithm, we found five distinct cellular composition clusters. Tumors unresponsive to anti-PD-1/CTLA-4 immunotherapy showed enrichment of a FoxP3^+^ T-cell-low TLS cluster after treatment. Additionally, cluster 5 (macrophage low) TLS were significantly higher after pre-operative immunotherapy, compared to untreated tumors. We also compared the immune cell composition and maturation stages between superficial (submucosal) and deeper TLS, revealing that superficial TLS had more pronounced T-helper cells and enrichment of early TLS than TLS located in deeper tissue. Furthermore, superficial TLS displayed a lower fraction of secondary follicle like TLS than deeper TLS. Taken together, our results provide a detailed quantitative overview of the tumor immune landscape in UC, which can provide a basis for further studies.

## 1 Introduction

Muscle-invasive urothelial cancer (UC) is an aggressive disease with limited treatment options that originates in the bladder and parts of the urinary tract. Although UC can be cured by resection of the bladder (cystectomy), recurrence rates are high and 5-year survival is only 60-70% for pT2N0 tumors, and even worse for high-risk patients having pT3-4aN0 (40-50%) or pTxN+ (10-35%) at cystectomy. Immune checkpoint inhibitors (ICIs) have changed the treatment paradigm in metastatic urothelial cancer. Currently, ICIs have been approved for the first-line and second-line treatment (1–5), and are being tested in the adjuvant and preoperative setting. In the PURE-01 trial (6) and ABACUS trial (7), preoperative pembrolizumab (anti-PD-1) and atezolumab (anti-PD-L1) were clinically tested in patients diagnosed with cT2-4N0 UC, respectively. These trials revealed promising pathological complete response (pCR) rates upon treatment with neo-adjuvant pembrolizumab and atezolizumab. However, pCR to ICI monotherapy was primarily found in patients having less extensive disease (cT2N0), whereas patients with more extensive disease (cT3-4N0) or loco-regional lymph node involvement (T2-4N+) showed only limited pCR to anti-PD1 or anti-PD-L1. Recent clinical studies testing combination strategies targeting PD-1/PD-L1 plus CTLA-4 in the metastatic setting found higher response rates than in trials testing anti-PD1 or anti-PD-L1 alone (8, 9). In the NABUCCO trial (10), preoperative ipilimumab plus nivolumab was tested in high-risk patients having locoregionally-advanced UC (cT3-4N0/cT2-4N1-3) without distant metastases. Histopathological examination showed that 58% of patients in NABUCCO had no remaining invasive disease (pT0 or CIS/pTa) after ipilimumab plus nivolumab (10). A study testing preoperative tremelimumab plus durvalumab in cT2-4N0 UC observed a pCR in 37.5% (pT0 or CIS) of patients having surgery, whereas the pCR rate was 31.7% in all patients analyzed (8).

Associations between ICI response and candidate biomarkers, such as PD-L1 immunohistochemistry and tumor mutational burden (TMB), have been observed in metastatic UC. These biomarkers are currently imperfect and lack sufficient predictive power for clinical utility (11, 12). In addition, comparison of biomarker findings across trials is complicated by variability in biomarker assays (i.e. PD-L1 assessment) and heterogeneity in tumor tissue used to assess biomarkers. In the preoperative setting, the pCR rate to pembrolizumab in the PURE-01 study was high in TMB-high and PD-L1-high (PD-L1 >10%; tumor plus immune cells combined) tumors (6), whereas no significant associations were found for TMB-high and PD-L1-high (PD-L1 >5% of immune cells) subgroups in anti-PD-L1 treated patients in ABACUS (7). Both studies found that baseline pre-existing CD8^+^ T-cell immunity based on high CD8 presence and interferfon-γ signaling was associated with pCR to ICI monotherapy. Qualification of immune phenotypes by CD8 immunohistochemistry showed that “immune desert” tumors in ABACUS were unresponsive to ICI (7). In sharp contrast, the clinical response to combination ICI in NABUCCO was independent of baseline CD8^+^ T-cell density by multiplex immunofluorescence and inflammatory signatures such as interferon-gamma, tumor inflammation and T-cell effector signatures (10). Similarly, baseline pre-existing CD8^+^ T-cell immunity did not differ between responders and non-responders to neo-adjuvant tremelimumab plus durvalumab (13), suggesting that the addition of anti-CTLA4 can induce responses in immunologically “cold” tumors.

Tertiary lymphoid structures (TLS) are ectopic lymph node formations that share functional features such as antigen presentation and B-cell activation with secondary lymphoid organs. TLS emerge upon chronic inflammatory stimuli in non-lymphoid tissues and can also be found in the tumor micro-environment. In an analysis of the presence of TLS, responders to tremelimumab plus durvalumab showed higher baseline TLS and B-cell abundance than non-pCR tumors. Intriguingly, baseline TLS and B-cell abundance did not differ between responders and non-responders in NABUCCO. However, both studies found that responders to combination ICI showed a higher TLS abundance in post-treatment tissue than non-responders (10, 13). Thus, conflicting results on baseline candidate biomarkers for immunotherapy response were found between comparable studies. The complex interplay between immune cells in the UC tumor-immune microenvironment and TLS is still poorly understood, hampering the discovery and development of novel cancer immunotherapy as well as predictive biomarkers for immunotherapy response, underscoring the urgent need to better characterize the tumor immune landscape in UC.

In this study, we employ quantitative multiplex immuno-fluorescence to assess the UC tumor-immune contexture in untreated and immunotherapy-treated tumors. We first provide a general overview of the UC tumor-immune microenvironment, followed by a more detailed assessment of the TLS immune composition in untreated and immunotherapy-treated tumors.

## 2 Results

### 2.1 Untreated Urothelial Cancer Demonstrates Heterogeneous Immune Cell Infiltration

**Figure 1:**
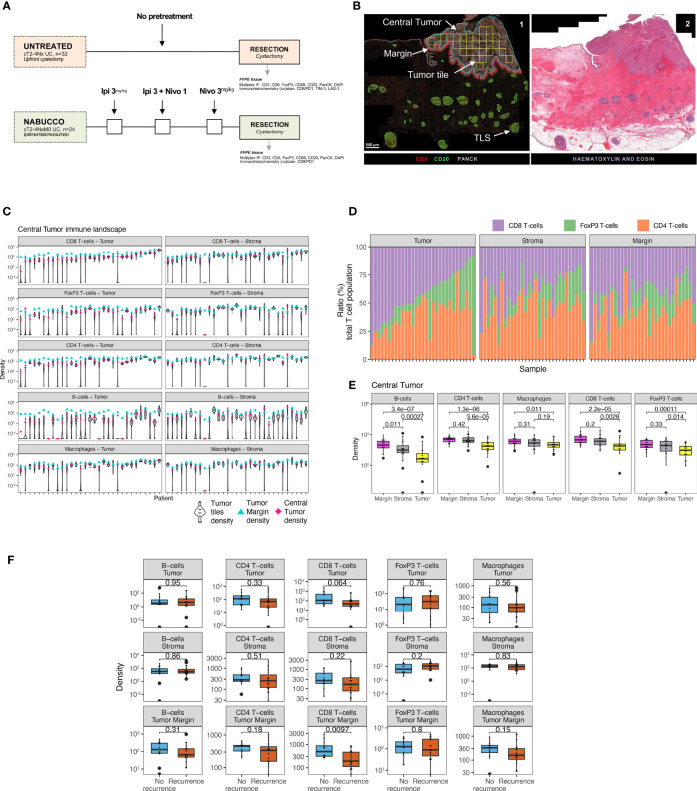
Untreated urothelial cancer demonstrates heterogeneous immune cell infiltration. **(A)** Cohort interventions and timepoints of tissue collection for biomarker analysis. **(B)** *1)* Example of annotated regions of interest in untreated urothelial cancer analyzed by multiplex immunofluorescence and HALO image analysis, involving annotations of central tumor (blue), central tumor tiles (yellow, n = 30 tiles per slide), tumor margin (red, 250 micrometers diameter and tertiary lymphoid structures (green). Central tumor area can be distinguished in tumor and stroma area by employing and training a tissue classifier. *2)* Corresponding H&E slides were consulted to support annotation of regions of interest. **(C)** Intratumoral and stroma immune subset densities per mm^2^ within tumor tiles (violin plots, n = 30 tiles per sample), whole tumor (pink) and tumor margin (cyan) for the untreated UC cohort (n = 31). The median immune subset densities and distribution across tumor tiles were analyzed by quantitative multiplex immunofluorescence. Samples were sorted by intratumoral CD8 T-cell central tumor density. **(D)** Relative abundance of T-cell subsets in the total T-cell population in central tumor tissue classes and tumor margin by multiplex IF (n = 31). Samples were sorted by CD8 T-cell ratio. **(E)** Immune subset densities per mm^2^ for tumor tissue regions in the untreated UC cohort (n = 31). **(F)** Intratumoral, stroma and tumor margin immune subset densities per mm^2^ for the combined untreated UC cohort (n = 31) between recurrence (n = 19) and non-recurrence (n = 12) groups. The boxplots from the panels display the median and 25th and 75th percentiles, and the whiskers expand from the hinge to the largest value not exceeding the hinge 1.5×Interquantile range. Unless otherwise stated, a two-sided Mann-Whitney test was used for the comparison between distributions. The p-value is presented in-between boxplots. No adjustments were implemented for multiple comparisons. IF, Immuno-fluorescence; FFPE, Formalin-fixed paraffin-embedded tissue; Ipi, Ipilimumab; Nivo, Nivolumab; TLS, Tertiary lymphoid structure.

To examine the UC immune context, we analyzed immune cell infiltration by multiplex immunofluorescence (IF) on whole-slide cystectomy tissue sections from untreated (n=32, **Table 1**) and ipilimumab (anti- CTLA-4) plus nivolumab (anti-PD1) treated (n=24, **Table 2**) UC patient cohorts (**Figure 1A** **)**. In the current study, cystectomy specimens obtained from NABUCCO are analyzed, while we previously (10) reported CD8^+^ and CD20^+^ immune cell presence in pretreatment biopsies. Additionally, we segmented tumor areas into various regions of interest. Our antibody panel allowed the quantitation of immune cells actively involved in anti-tumor immunity and response, such as B-cells (CD20^+^), macrophages (CD68^+^) and distinct CD3^+^ T-cell populations. CD3^+^ T-cell populations were further specified by expression of CD8 or FoxP3, resulting in CD8 T-cells (CD3^+^CD8^+^), FoxP3 T-cells (CD3^+^FoxP3^+^) and CD4 T-cells (CD3^+^CD8^-^FoxP3^-^), a non-CD8^+^/FoxP3^+^ T-cell population which is likely to involve primarily CD4 T-cells. CD3^+^FoxP3^-^CD8^-^ was thus used as an approximation of CD4^+^ T-cells to make the manuscript easier to read. CD4 IF was not used in our multiplex panel given the expression of CD4 on other immune cells (including macrophages and dendritic cells) when using CD4 antibodies in our pilot studies. Immune cells were separately quantified for tumor and stroma areas within the central tumor and square grids were computed for spatial sampling to assesses heterogeneity of immune subsets within tumors (**Figure 1B** and **Supplementary Methods 1** **)**. We additionally quantified immune cell abundance in the tumor margin and TLS. The tumor margin was annotated from the outermost edge of the invasive tumor, with an extend of 250µm **(** **Supplementary Methods 1** **)**. To promote readability, immune cell labels and not markers are reported throughout the results.

**Table 1:** Untreated cohort characteristics.

Baseline characteristics	Total (n = 31)
Male sex, n (%)	24 (77%)
Median age – years [range]	64.79 [45.7, 78.7]
Pathological T stage, (%)	
pT1-4/pTis/pTaN0M0	20 (65%)
pT3-4N1-2M0	11 (35%)
Histology, (%)	
Urothelial Carcinoma	29 (94%)
Urothelial Carcinoma and Small cell carcinoma	1 (3%)
Urothelial Carcinoma and Squamous differentiation	1 (3%)
Adjuvant treatment, (%)	
No adjuvant treatment	25 (81%)
Adjuvant chemotherapy	2 (6%)
Adjuvant radiotherapy	3 (10%)
Adjuvant chemotherapy and adjuvant radiotherapy	1 (3%)

**Table 2:** Ipilimumab plus nivolumab treated cohort (NABUCCO Cohort 1) characteristics.

Study population characteristics	Total (n = 24)
Male sex, n (%)	18 (75%)
Median age – years [range]	65 [50, 81]
Baseline clinical T stage, (%)	
cT3-4N0M0	14 (58%)
cT3-4N1	5 (21%)
cT2-3N2M0	5 (21%)
Post-treatment clinical stage, (%)	
ypT0/pTa/pTisN0M0/Mx	14 (58%)
ypT2-3N0M0	2 (8.5%)
ypT0-4N1M0	6 (25%)
ypT3N2-3M0	2 (8.5%)
Immunotherapy cycles, (%)	
2	6 (25%)
6	18 (75%)

We first examined immune cell infiltration by multiplex IF for tumor and stroma areas to provide a comprehensive overview of the UC immune contexture and assess intratumor heterogeneity. We observed that the median density of immune subsets varied greatly across the untreated tumor cohort, particularly for B-cells, FoxP3 T-cells and CD8 T-cells **(** **Figure 1C** **)**. Variable intratumoral heterogeneity existed for specific immune cells upon a comparison of separate tiles in the computed square grid **(** **Figure 1C** **).** Next, we examined the relative abundance of T-cell subsets in the total T-cell population. We found that the fraction of CD4 T-cells was highly heterogeneous across tumors in the untreated cohort **(** **Supplementary Figure 1A** **)**. Further explorative analysis revealed that tumors having a low CD8 T-cell ratio demonstrated a higher proportion of FoxP3 T-cells in tumor **(** **Figure 1D** and **Supplementary Figure 1B** **)**. We then compared the immune cell density between central tumor regions and the tumor margin. A significantly higher presence of immune cells was found in tumor margins when compared to the tumor region (p<0.02 **Figure 1E**). In non-recurring tumors, the tumor margins displayed a significantly higher CD8 T-cell presence than in recurring tumors **(**p=0.0097, **Figure 1F** **)**, while immune cell presence in tumor and stroma did not inform clinical outcome in untreated tumors. In conclusion, the UC immune landscape is heterogeneous between tumors, and pronounced immune infiltration is found in the UC tumor margin (7, 14).

### 2.2 Urothelial Cancer Immune Phenotypes Show Distinct Patterns of Cytotoxic T-Cell Exclusion in the Stroma and Tumor Margin

**Figure 2:**
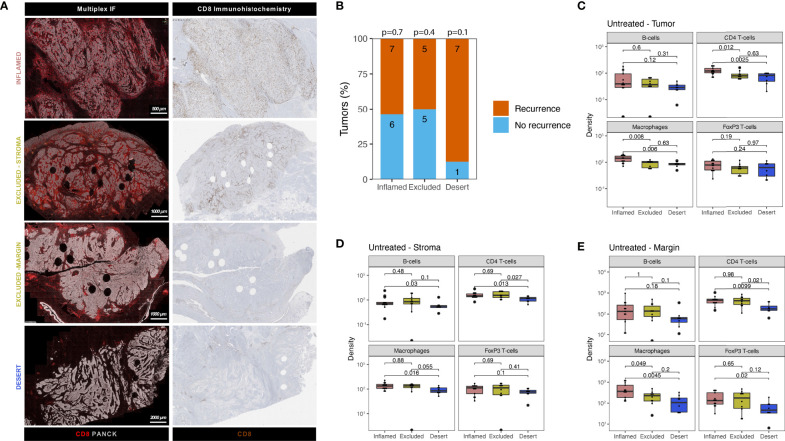
Urothelial cancer immune phenotypes display a varying abundance of immune cells and distinct patterns of cytotoxic T-cell exclusion in stroma and tumor margins. **(A)** Examples of tumor immune phenotypes by multiplex immunofluorescence and CD8 immunohistochemistry in untreated urothelial cancer **(B)**. Proportion of patients having recurrence (n = 19) and no recurrence (n = 12) stratified by immune phenotype for the untreated UC cohort. The group size is indicated on each bar. A Fisher’s exact test was implemented on a 2x2 contingency table between recurrence and immune phenotype (i.e. Desert *vs* No Desert) for each phenotype. The p-value for each phenotype is indicated at the top of each bar. All statistical tests were two-sided. C-E. Comparison of immune subset densities per mm^2^ in central tumor parenchyma **(C)**, central tumor stroma **(D)** and tumor margin **(E)** between inflamed (n = 13), excluded (n = 10) and desert (n = 8) tumors by quantitative multiplex IF. The boxplots from the panels display the median and 25^th^ and 75^th^ percentiles, and the whiskers expand from the hinge to the largest value not exceeding the hinge 1.5×Interquantile range. Unless otherwise stated, a two-sided Mann-Whitney test was used for the comparison between distributions. The p-value is presented in-between boxplots. No adjustments were implemented for multiple comparisons. IF, immunofluorescence.

CD8 T-cell tumor infiltration patterns can be segregated into three immune phenotypes (“immune-inflamed”, “immune-excluded” and “immune-desert”) of pre-existing tumor-immunity (15). Previous studies found that these distinct immune phenotypes harbor prognostic relevance (16) and predictive value (17, 18) for an immunotherapy response, including in UC (7, 14). Currently, limited knowledge exists on the presence of distinct immune subsets beyond cytotoxic T-cells across CD8-based immune phenotypes in UC, while their presence may impact CD8 effector function and the extend of CD8 tumor-immunity. Using multiplex IF, immune phenotypes (**Figure 2A**) were classified based on CD8 T-cell density (**Supplementary Methods 1.2**) in the tumor and stroma compartment and the tumor margin in the untreated UC cohort. We first explored the distribution of tumor immune phenotypes in the untreated cohort and assessed possible correlations with prognosis for “inflamed”, “excluded” and “desert” tumors separately. In line with results in the ABACUS study (7), “immune-inflamed” (42%) tumors were most abundant in our cohort, whereas 32% and 26% of tumors exhibited the “excluded” and “desert” phenotype, respectively. The separate tumor immune phenotypes did not inform recurrence outcome in the untreated cohort (**Figure 2B**), although tumors qualified as “immune-desert” showed a high recurrence rate (87.5%, p=0.1). Next, we explored the immune composition in tumor subgroups qualified as “immune-inflamed”, “immune-excluded” and “immune-desert” based on CD8-based immune phenotypes. Intratumoral immune cell densities were generally higher in “inflamed” tumors compared to “excluded” and “desert” tumors, as shown for the significantly higher macrophages compared to “desert” tumors (p=0.006. **Figure 2C**). In the stroma compartment, immune cell densities were lowest in “desert” tumors, as shown for the significantly lower CD4 T-cells when compared to “excluded” (p=0.027) and “inflamed” tumors (p=0.013) (**Figure 2D**). Interestingly, FoxP3 T-cells were an exception, as these cells were similar across immune phenotypes in absolute density and higher as a percentage of total T-cells in “desert” tumors, compared to “inflamed” tumors (p=0.037, **Supplemental Figure 2A**). Macrophage abundance in tumor margins of “inflamed” tumors was significantly higher than in “excluded” (p=0.049) and “desert” (p=0.005) tumors, (**Figure 2E**).

### 2.3 Markers of T-Cell Exhaustion in Untreated and Immunotherapy Treated UC

**Figure 3:**
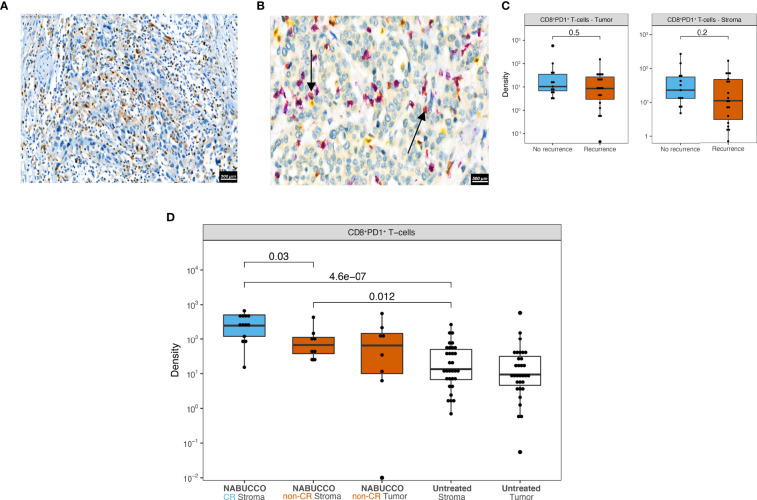
Markers of T-cell exhaustion in untreated and immunotherapy treated UC. **(A)** Representative tumor analyzed by TIM-3 immunohistochemistry. **(B)** Representative tumor analyzed by CD8 (purple) PD1 (yellow) co-stainings in untreated UC, showing CD8^+^PD1^+^ (red) T-cells marked by black arrows. **(C)** CD8^+^PD-1^+^ cell densities in central tumor regions stratified by recurrence outcome in untreated UC (n_Recurrence_ = 19, n_No recurrence_ = 13). **(D)** CD8^+^PD-1^+^ cell densities in central tumor regions in untreated (n_Untreated Stroma_ = 32, n_Untreated Tumor_ = 32) and immunotherapy treated cystectomies (n_NABUCCO CR Stroma_ = 13, n_NABUCCO non-CR Stroma_ = 8, n_NABUCCO non-CR Tumor_ = 8). Non-significant comparisons, as well as comparisons between tumor and stroma regions, were excluded from the plot. The boxplots from the panels display the median and 25^th^ and 75^th^ percentiles, and the whiskers expand from the hinge to the largest value not exceeding the hinge 1.5×Interquantile range. Unless otherwise stated, a two-sided Mann-Whitney test was used for the comparison between distributions. The p-value is presented in-between boxplots. No adjustments were implemented for multiple comparisons. CR, complete response; non-CR, no complete response.

Exhausted CD8 T-cells are characterized by impaired effector function and sustained expression of immune inhibitory checkpoints such as TIM3, LAG3 and PD1 (19). Immuno-therapies targeting these checkpoints demonstrate promising therapeutic potential in several studies (20–26), presumably by reinvigorating exhausted T-cells. Given the implication of T-cell exhaustion as a target of immunotherapy, we employed immunohistochemistry in our untreated cohort to examine the expression of TIM3 and LAG3, as well as co-expression of CD8 and PD1. In untreated tumors, we observed considerable TIM-3 expression (example image in **Figure 3A**) on tumor-infiltrating lymphocytes (15% median positivity, range 5%-30%, **Supplementary Figure 3A**) in most central tumors, as well as in lymph nodal T-cell zones in rare cases having perivesical lymph nodes adjacent to the central tumor (**Supplementary Figure 3B**). In contrast to TIM-3, expression of LAG-3 was virtually non-existent in untreated tumors (**Supplementary Figure 3C**), as illustrated in **Supplementary Figure 3D**. Following CD8/PD1 co-staining, an algorithm was trained (**Supplementary methods 1.3**), based on a similar approach as in colorectal cancer (20), to assess CD8^+^PD1^+^ T-cells in tumor and stroma. CD8^+^PD1^+^ T-cells were clearly present in untreated UC, as shown in **Figure 3B**. Upon quantitation, we found that CD8^+^PD1^+^ T-cell abundance in tumor and stroma did not inform recurrence (**Figure 3C**). We then examined CD8^+^PD1^+^ T-cells in NABUCCO tumors having complete response (CR, qualified as pCR or CIS/pTa) and non-CR following ipilimumab plus nivolumab. CD8^+^PD1^+^ T-cells were enriched irrespective of response compared to untreated cystectomies, whereas CD8^+^PD1^+^ T-cells were highest in tumors achieving CR to immunotherapy (**Figure 3D**). Altogether, TIM-3 was highly expressed on lymphocytes and abundant CD8^+^PD1^+^ T-cells were found in cystectomies, particularly following immunotherapy, in both responders and non-responders.

### 2.4 Urothelial Cancer TLS Display Distinct Cellular Composition Clusters and Checkpoint Inhibitor-Induced Changes

**Figure 4:**
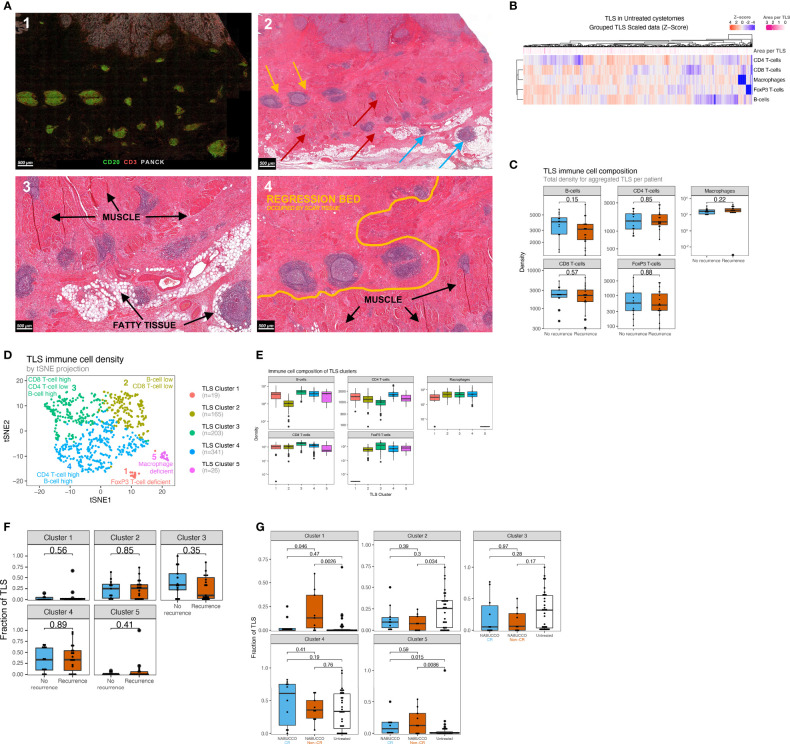
Urothelial cancer displays distinct TLS clusters and differences in treatment effect on TLS composition between responders and non-responders. **(A)** 1) Multiplex immunofluorescence example showing substantial peritumoral TLS formation. 2) Corresponding hematoxylin and eosin stain, showing TLS formation in muscle (red arrow), fatty tissue (blue arrow) and fibroinflammatory regression bed (yellow). 3). Close-up image of A2, showing TLS formation around muscle, fatty tissue and in regression bed. 4) Regression bed TLS and depositions of scar tissue in areas previously harboring muscle suggest that pre-existing invasive tumor has been cleared and replaced by scar tissue, suggesting pre-existing antitumor immunity. **(B)** Heatmap showing the variability of immune cell density in untreated UC TLS. Each column represents an individual TLS (n=754) from n=32 patients. Z-score high expression levels (red) and low expression levels (blue) and varying TLS size (pink) are indicated for each TLS. **(C)** TLS immune subset densities per mm^2^, stratified by recurrence outcome groups (n_Recurrence_=19, n_No Recurrence_=13). **(D)** Clustering map upon computing a trained k-means model using 754 untreated TLS from 32 unique patients of the untreated cohort (Median 16.5 TLS per patient, Mean 24 TLS per patient, Materials and Methods 3). Each TLS type is assigned a color label and an interpretation. **(E)** Abundance of Immune subsets per mm^2^ for each TLS cluster. TLS clusters are depicted in distinct colors. (n_Cluster1 =_ 19, n_Cluster 2 =_ 165, n_Cluster 3 =_ 203, n_Cluster 4 =_ 341, n_Cluster 5 =_ 26) **(F)**. Comparisons of TLS relative area per cluster based on multiplex immunofluorescence between non-recurring tumors (n = 13) and tumors having recurrence (n = 19). **(G)** Comparisons of post-treatment TLS cluster fractions between untreated tumors (n = 32) and complete-responders (n = 10) and non-responders (n = 9) in NABUCCO. The boxplots from the panels display the median and 25th and 75th percentiles, and the whiskers expand from the hinge to the largest value not exceeding the hinge 1.5×Interquantile range. Unless otherwise stated, a two-sided Mann-Whitney test was used for the comparison between distributions. The p-value is presented in-between boxplots. No adjustments were implemented for multiple comparisons. CR, complete response; non-CR, no complete response; TLS, Tertiary lymphoid structures.

In many cancers, the immune landscape exhibits highly organized B-cell-rich clusters related to TLS formation. The presence of TLS has been associated with favorable clinical outcomes in untreated and treated malignancies (13, 27–29), whereas other studies found no correlation or immuno-suppressive TLS function (30–33). We hypothesized that heterogeneity in TLS immune composition might impact anti-tumor-immunity and patient outcome in the untreated and treated setting. We employed multiplex IF to assess the cellular composition of TLS and associations with clinical outcome in our untreated cohort. TLS were automatically annotated by a trained algorithm and manually revised when needed. In total, 754 TLS aggregates were identified in untreated tumors mainly found around the muscularis propria regions, fatty tissue and fibroinflammatory regression beds (**Figure 4A**). TLS often co-localized with nerve bundles as confirmed on the corresponding H&E slide (**Supplementary Figure 4A**). Following TLS assessment by multiplex IF, the majority of untreated tumors showed notable TLS presence, but no differences in TLS abundance were observed between recurrence groups (**Supplementary Figure 4B**). Upon quantitative analysis, TLS revealed a heterogeneous cellular immune composition, accompanied by strong variations in TLS size between TLS in untreated tumors (**Figure 4B**). No differences were found for immune subset density in aggregated TLS between recurrence groups (**Figure 4C**). As limited knowledge exists on TLS immune architecture and how immune composition impacts the clinical outcome, we grouped TLS based on immune cell density and their relative abundance in untreated tumors using a k-means clustering algorithm. We identified five distinct TLS clusters in untreated tumors (**Figure 4D**), characterized by varying abundance of immune cells (**Figure 4E**), whereas TLS cluster presence was balanced between immune phenotype subgroups (**Supplementary Figure 4C**). No differences were observed for TLS cluster abundance between outcome groups (**Figure 4F**) in untreated UC. Next, the relative abundance of TLS clusters was compared between untreated tumors and anti-PD-1/CTLA-4 treated tumors to examine how immunotherapy impacts these TLS clusters. In NABUCCO non-responders, cluster 1 (FoxP3 T-cell low) TLS were significantly enriched when compared to untreated tumors or NABUCCO responders (**Figure 4G**). Furthermore, cluster 5 (macrophage low) TLS were significantly higher in NABUCCO (non-CR or CR) tumors compared to untreated tumors (**Figure 4G**). These findings suggest that UC displays distinct TLS clusters that change in cellular composition upon immunotherapeutic treatment.

### 2.5 Discrepant TLS Patterns and Variable Expression of CD4 T-Cells Between Superficial and Deeper TLS in Urothelial Cancer

**Figure 5:**
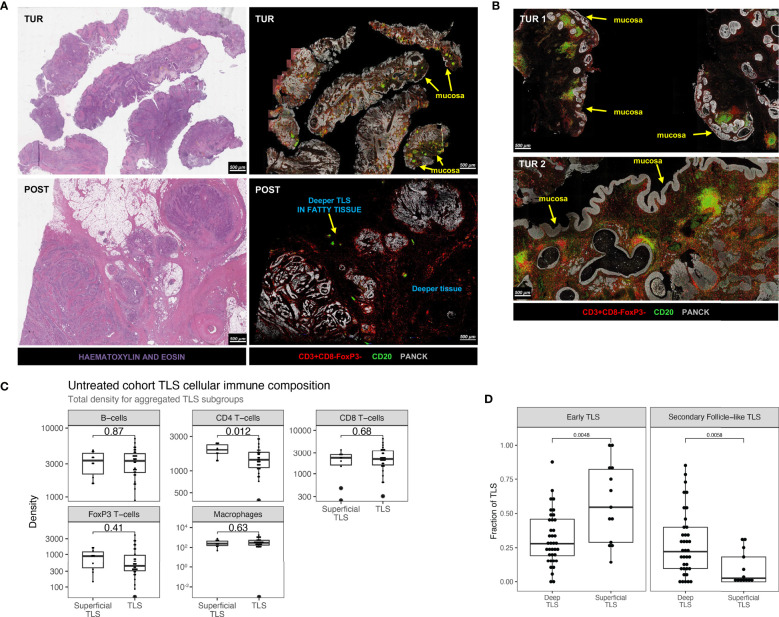
Discrepant TLS patterns and variable expression of T-helper cells between superficial and deeper TLS in urothelial cancer. **(A)** Example of TLS abundance in baseline TUR and post-treatment cystectomy by multiplex immunofluorescence and hematoxylin and eosin stain in a non-responding patient in NABUCCO. Baseline TUR tissue shows a higher TLS presence than in the post-treatment specimen, particularly in submucosal regions. **(B)** Two different TUR examples showing TLS that display pronounced CD4 T-cell presence. **(C)** Comparison of TLS aggregated immune cell density (counts per mm^2^) between superficial (n = 10 patients) and deeper TLS (n = 30 patients). **(D)** TLS maturation states quantified by CD21-expressing Follicular Dendritic Cell networks and CD23+ Germinal center zones by multiples IF (Materials and Methods). Quantifications were done on 40 untreated UC cystectomies (18 patients from the original untreated cohort (**Figure 1A**) and 22 additional untreated UC cystectomies, **Supplementary Table 1**). Fraction of TLS maturation states are depicted for Deep TLS (n = 37) and Superficial TLS (n = 13), for Early TLS (germinal center negative) and Secondary Follicle-like TLS (germinal Center positive). 27 patients had only Deep TLS present, 3 patients had only Superficial TLS present, and 10 patients had both Superficial and Deep TLS present. The boxplots from the panels display the median and 25th and 75th percentiles, and the whiskers expand from the hinge to the largest value not exceeding the hinge 1.5×Interquantile range. Unless otherwise stated, a two-sided Mann-Whitney test was used for the comparison between distributions. The p-value is presented in-between boxplots. No adjustments were implemented for multiple comparisons. TLS, Tertiary lymphoid structures; TUR, Transurethral Resection; UC, Urothelial Carcinoma.

Although pretreatment B-cell and TLS enrichment has been associated with favorable clinical outcomes and immunotherapy response, other studies reported no positive associations (10, 13), suggesting that B-cells and TLS can have opposite roles. In NABUCCO, we previously found that immature TLS, B-cells, and genes associated with B-cell proliferation and plasma cells were enriched in pretreatment biopsies in non-CR tumors, compared to CR tumors (10). Conversely, a study testing preoperative tremelimumab plus durvalumab in UC reported higher pretreatment TLS and B-cells in responders (13). As other stimuli have been shown to induce TLS (31, 34, 35), we hypothesized that a subset of TLS may be unrelated to anti-tumor immunity, particularly in pretreatment tissue obtained by transurethral resection (TUR, debulking of a tumor from the luminal layer of the bladder). TUR biopsies primarily collect superficial tissue that is highly exposed to urinary toxins, microbial pathogens (especially in the presence of a bladder tumor) and inflammatory mediators (**Supplementary Figure 5A, B**). These TLS could cloud the tumor-associated TLS analysis, particularly in superficial parts of the tumor. To examine this, we explored whether TLS composition in superficial regions differed from TLS in deeper tissue regions. In line with quantitated results in our previous report (10), a high TLS presence was observed in NABUCCO pretreatment TUR, especially in non-CR tumors, while TLS abundance was limited in their corresponding post-treatment tissues (**Figure 5A**). TLS abundance in pretreatment TUR was particularly high in the urothelial submucosa (**Figure 5B**). TLS present in the urothelial submucosa (Superficial TLS) were characterized by pronounced CD4 T-cell presence, whereas deeper TLS showed only limited CD4 T-cell contribution to the immune cell composition (**Figure 5B**). The predominant abundance of superficial TLS was also found in a subset of post-treatment specimens from NABUCCO (**Supplementary Figure 5C**) and untreated tumors (**Supplementary Figure 5D**), further supporting the existence of a distinct TLS population in superficial tissue. Next, we stratified superficial and deep TLS in untreated UC to compare TLS composition and the relative abundance of TLS clusters. In untreated tumors, superficial TLS showed a significantly higher CD4 T-cell presence (p=0.012, **Figure 5C**), which is in line with our visual observations. Next, we quantified TLS maturation stages for superficial and deep TLS using a 7-plex multiplex immunofluorescence panel on a separate, larger cohort (n=40, involving 20 patients from the original untreated cohort, **Supplementary Table 1**). Upon assigning TLS maturation, we found that superficial TLS displayed a higher fraction of early TLS and lower germinal center positive TLS when compared to deeper TLS (p=0.001 and p=0.01, respectively **Figure 5D**).

Altogether, our findings suggest that superficial TLS may be compositionally different from deeper TLS. These observations could impact the approach to immune biomarkers in UC and provides the rationale to dissect TLS populations further and study their precise role in anti-tumor immunity in the UC tumor-immune microenvironment.

## 3 Discussion

The introduction of ICI changed the treatment landscape of UC. Despite recent successes, a substantial proportion of patients do not respond to immunotherapy (36, 37). As the biology driving anti-tumor immunity is still poorly understood, the characterization of the tumor immune contexture is critical to broaden our understanding of the immune landscape to ultimately improve immunotherapeutic treatment of UC patients (11).

The aim of our study was to characterize the immune landscape in tumor, stroma and TLS using computational analysis of multiplex IF. We started with a general overview of the UC immune landscape and observed substantial variation in immune subset presence across untreated tumors. Immune cells were more abundantly present in the tumor margin, compared to tumor and stroma. In previous UC immune biomarker studies, the tumor margin immune infiltrate was not specifically reported (6) or incorporated into the immune phenotype classification system (7, 14). In other cancer types such as colorectal cancer, breast cancer and melanoma, tumor margins have been extensively used for immune phenotype assessment (38). In UC, T-cell exclusion by TGF-beta signaling has been proposed as a mechanism of resistance by excluding T-cells, emphasizing the importance of incorporating the tumor margin compartment in biomarker assessment in UC.

Tumor-specific T-cells can be re-activated through blocking immune inhibitory checkpoints (20–26). We observed high TIM-3 expression and abundant CD8^+^PD1^+^ T-cell presence in UC. CD8^+^PD1^+^ T-cells were enriched upon immunotherapy, and surprisingly, also in immunotherapy non-responders. These data suggest that, despite the immune system being able to mount an anti-cancer response upon checkpoint blockade, resistance mechanisms beyond the CTLA-4 and PD-1 checkpoints may limit cytotoxic T-cell effector function and tumor elimination in these cases. A further dissection of the tumor-immune landscape in non-responders is crucial to identify the resistance mechanism limiting the efficacy of checkpoint blockade.

In this study, we found that UC exhibits distinct TLS clusters with varying cellular composition. We observed that upon CTLA-4/PD-1 blockade, the fraction of TLS clusters 1 (FoxP3 T-cell low) was enriched in non-responding tumors when compared to untreated tumors and responding tumors. Tregs are generally believed to have immune-suppressive functions, though limited data exist on the function of these cells within TLS. In a lung cancer mouse model, Treg presence in TLS was associated with a suppressed T-cell function (39). Studies in colorectal cancer (40) and melanoma (41) found no correlation between Treg presence in TLS and patient survival. A possible reason for the enrichment of Treg-low TLS may be a direct therapeutic effect of anti-CTLA4, depleting Tregs in TLS. Despite Treg depletion, these tumors did not respond, suggesting that other causes for resistance might be present in these tumors (11, 42).

Generally, TLS in the tumor-microenvironment is considered tumor-associated. Our findings suggest that superficial TLS may define a distinct TLS category in UC that may not be tumor-responsive. Superficial bladder tissue may exhibit immune features (e.g., TLS) unrelated to anti-tumor immunity, given the high exposure to urinary toxins or microbial pathogens, especially in the presence of a bladder tumor disrupting the mucosal barrier. We found that these superficial TLS had a higher density of CD4 T-cells. The proportion of secondary follicle-like TLS, which are required for the prognostic benefit of TLS in other cancer types (27, 43), was significantly lower in superficial TLS compared to deep TLS. Given the similar characteristics, we hypothesize that superficial TLS may be related to Hunner-type interstitial cystitis, an idiopathic inflammatory disease characterized by submucosal lymphocytic pan-cystitis, lymphoid aggregates (Hunner lesions) with varying maturation stages (44) and expression of follicular T-helper cell markers (45). In addition, a recent study showed that Hunner-type interstitial cystitis was associated with enrichment of B-cell receptor signaling genes and B-cell clonal expansion (46). In line with these findings, we previously found that immature TLS, B-cells and genes associated with B-cell proliferation and plasma cells were enriched in baseline TUR tissue in non-CR tumors (10). These discrepant findings in NABUCCO may be explained by the presence of tumor-unrelated TLS such as Hunner-type aggregates in the TUR samples. One can even speculate that high numbers of superficial TLS indicate prominent chronic inflammation with adverse effects on anti-tumor immunity, explaining the association with non-response. This hypothesis needs further testing. In biomarker assessments, the presence of submucosal TLS may possibly enrich B-cell and TLS levels independent of anti-tumor immunity, particularly in TUR (which removes superficial layers) and smaller biopsies. In non-UC patients, the prevalence of interstitial cystitis is 0.5% in the western world (47). No data exists on interstitial cystitis in muscle-invasive bladder cancer, because of the prognostic impact of bladder cancer and overlapping locoregional symptoms.

The strengths of the current study are the comprehensive computational analysis and the automated nature of our assessments, enabling 1) in-depth analysis of the tumor bed, and 2) systematic assessment of tertiary lymphoid structure’s immune architecture in untreated and ICI treated tumors. Combined, our study provides a unique overview of the UC immune landscape. Limitations include the limited sample size, which precluded robust assessment of associations with outcome, and the number of immune markers profiled, which limited insight into the functional relevance of immune cells. Further limitations include the retrospective nature of our study and the risk of overinterpretation due to multiple testing.

In conclusion, our study provides a comprehensive overview of the tumor immune landscape and architecture of TLS in UC. We established distinct TLS clusters based on their cellular compositions. Compared to untreated tumors, TLS clusters showed a distinct immune cell composition in anti-CTLA-4/PD-1 ICI treated tumors. In addition, we identified a superficial TLS population, characterized by more pronounced CD4 T-cell expression than deeper TLS. The relevance of the superficial TLS population for antitumor immunity is currently unknown and warrants further investigation.

## 4 Materials and Methods

### 4.1 Study Cohort Characteristics

Tumors were obtained from untreated patients and a prospective clinical trial testing the efficacy of preoperative ipilimumab (anti-CTLA-4) plus nivolumab (anti-PD-1) (NABUCCO: NCT03387761). In NABUCCO, a total of 24 patients with stage III resectable urothelial cancer (cT3-4aN0M0 and cT1-4aN1-3M0) were treated with preoperative ipilimumab 3 mg/kg (day 1), ipilimumab 3 + nivolumab 1 mg/kg (day 22), and nivolumab 3 mg/kg (day 43) followed by surgical resection. In the untreated cohort (n=31), patients had upfront cystectomy without prior systemic therapy following diagnosis of muscle-invasive carcinoma in pretreatment transurethral resection (TUR) specimen. Cystectomy specimens were preferred over TUR, given that TUR specimens provide a limited overview of the overarching tumor contexture, as shown in **Supplementary Figure 5**. The NABUCCO trial was approved by the institutional review board of the Netherlands Cancer Institute and was executed in accordance with the protocol and Good Clinical Practice Guidelines defined by the International Conference on Harmonization and the principles of the Declaration of Helsinki. Use of the cohort of untreated cystectomies was approved by the NKI-AVL institutional research board, following national regulations. Archival FFPE tumor tissue cystectomy specimens were used for immunohistochemistry and multiplex immunofluorescent analysis. Non-recurring patients and patients having recurrence were compared for explorative biomarker analysis. In NABUCCO, tumors with complete response (CR, defined as pCR, pTis or pTaN0) were compared to non-CR tumors for biomarker exploration. We included non-invasive disease in the CR definition, which is generally believed to be cured by surgery.

### 4.2 Multiplex Immunofluorescence Analysis and Immunohistochemistry

#### 4.2.1 Multiplex Immunofluorescence of CD8/CD4 T-Cells, B-Cells, Macrophages, and B-Cells

Analysis of immune cell subsets was performed by multiplex Immunofluorescence (IF) technology using an automated multiplex staining on a Discovery Ultra Stainer. Prior to multiplex staining, 3µm slides were cut on DAKO Flex IHC slides. Slides were then dried overnight and stored in +4°C. Before a run was started tissue slides were baked for 30 minutes at 70°C in an oven. Opan 7-color manual IHC kit (50 slides kit, Perkin Elmer, cat NEL81101KT) was used for staining. The protocol was initiated by heating the FFPE cuts for 28 minutes at 75°C, followed by dewaxing with Discovery Wash using the standard setting of 3 cycles of 8 minutes at 69°C. Cell Conditioning 1 (CC1, Ventana Medical Systems) was performed with Discovery CC1 buffer for 32 minutes at 95°C, after which Discovery Inhibitor was applied for 8 minutes to block endogenous peroxidase activity. Specific markers were detected consecutively on the same slide with the following antibodies, which included anti-CD3 (SP7, Cat RM-9107-S, ThermoScientific, 1/400 dilution 1 hour at RT), anti-CD8 (Clone C8/144B, Cat M7103, DAKO, 1/100 dilution 1 hour at RT), anti-CD68 (Clone KP1, M0814, Dako, 1/500 dilution, 1 hour at RT), anti-FoxP3 (clone 236A/47, Cat ab20034, Abcam, 1/50 dilution, 2 hours at RT), anti-CD20 (Clone L26, cat M0755, Dako, 1/500 dilution, 1 hour at RT) anti-PanCK (Clone AE1AE3, Cat MS-343P, Thermo Scientific, 1/100 dilution, 2 hours at RT).

Each staining cycle consisted of four steps: Primary Antibody incubation, Opal polymer HRP Ms+Rb secondary antibody incubated for 32 minutes at RT, OPAL dye incubation (OPAL520, OPAL540, OPAL570, OPAL620, OPAL650, OPAL690, 1/50 or 1/75 dilution as appropriate for 32 minutes at RT) and an antibody denaturation step using CC2 buffer for 20minutes at 95°C. Cycles were repeated for each new antibody to be stained. At the end of the protocol slides were incubated with DAPI (1/25 dilution in Reaction Buffer) for 12 minutes. After the run was finished slides were washed with demi water and mounted with Fluoromount-G (SouthernBiotech, cat 0100-01) mounting medium. After staining, imaging of the slides was done using the Vectra 3.0 automated imaging system (PerkinElmer). First, whole slide scans were made at 10x magnification. After selection of the region of interest, multispectral images were taken at 20x magnification. Library slides were created by staining a representative sample with each of the specific dyes. Using the InForm software version 2.4 and the library slides the multispectral images were unmixed into 8 channels: DAPI, OPAL520, OPAL540, OPAL570, OPAL620, OPAL650, OPAL690 and Auto Fluorescence and exported to a multilayered TIFF file. The multilayered TIFF’s were fused with HALO software (Indica Labs, v2.3). Analysis was done using HALO (Indica Labs, v2.3) image analysis. Pragmatic definitions and delineation of tumor regions in a spatial context are described in **Supplementary Methods 1.1**. Tumor and stroma regions were classified by HALO automated tissue segmentation. Quantitative assessment of central tumors was assessed in 31/32 patients, as one slide involved insufficient tumor material for appropriate assessment but did involve notable TLS (**Supplementary Methods 1.4**).

#### 4.2.2 Multiplex Immunofluorescence of Tertiary Lymphoid Structures Maturation States

TLS maturation was analyzed in tissue sections by 7-plex multiplex IF as previously described (Silina et al., 2018, Springer Protocols) (48). Briefly, tissue sections were deparaffinized, rehydrated and retrieved all in one step using the Trilogy buffer (CellMarque) for 10 min at 110°C in a pressure cooker. The following antibodies and dilutions were used for a 7-plex IF; CD21 (1:5000, clone 2G9 Leica), DC-LAMP (1:1000, clone 1010E1.01, Dendritics), CD23 (1:1000, clone SP3, Abcam), PNAd (1:5000, clone MECA-79, Biolegend), CD20 (1:5000, clone L26, Dako), CD3 (1:1000, clone SP7, ThermoScientific) and 200x magnified images were acquired by Vectra 3.0 multispectral microscope (PerkinElmer/Akoya). Area segregation was done by Inform tissue segmentation algorithm of the Inform software (Akoya).

TLS maturation stages were defined by the presence or absence of CD21^+^ Follicular Dendritic cells (FDC) networks and CD23^+^ Germinal Center (GC) cells in dense CD20^+^ B-cell regions. Proportions of early TLS (no FDCs, no GC), primary follicle-like (PFL) TLS (has FDCs but no GC) and secondary follicle-like (SFL) TLS were determined as fractions out of all analyzed TLS for each patient.

#### 4.2.3 Staining of TIM3, LAG3, and Co-Staining of CD8 and PD1

Stainings and co-stainings were performed by immunohistochemistry. Prior to the staining, 3µm sections were cut and dried overnight and subsequently transferred to Ventana Discovery Ultra autostainer. Briefly, paraffin sections were cut at 3 µm, heated at 75°C for 28 minutes, and deparaffinized in the instrument with EZ prep solution (Ventana Medical Systems). Heat-induced antigen retrieval was carried out using Cell Conditioning 1 (CC1, Ventana Medical Systems) for 64 minutes at 95°C. For the detection of TIM3, the clone D5D5R (Cell Signaling) was used (1/200 dilution, 1 hour, 370°C), and for the detection of LAG3, the clone 11E3 (1/50 dilution, 1 hour at 370°C, AbCam). The bound antibodies were detected using either Anti-Rabbit HQ (Ventana Medical Systems), 12 minutes at 37°C (TIM-3) or anti-mouse HQ (Ventana Medical Systems) for 12 minutes at 37°C (LAG-3) followed by Anti-HQ HRP (Ventana Medical Systems) for 12 minutes at 37°C and ChromoMap DAB Detection (Ventana Medical Systems). Slides were counterstained with Hematoxylin and Bluing Reagent (Ventana Medical Systems). For untreated tumors, the percentage of TIM-3 and LAG-3 expression on lymphocytes tumors was scored upon visual inspection of digital slides in Slidescore by a pathologist.

For the co-staining of PD-1 (yellow) and CD8 (purple), the protocol was adjusted. Detection of PD-1 was done using the antibody clone NAT105 (Ready-to-Use, 32 minutes at 37°C, Roche Diagnostics) in the first sequence. Visualization of the PD-1-bound antibody was done using anti-mouse NP (Ventana Medical Systems) for 12 minutes at 37°C, and subsequent anti-NP AP (Ventana Medical Systems) for 12 minutes at 37°C followed by the Discovery Yellow Detection Kit (Ventana Medical Systems). In the double-stain second sequence, CD8 was detected using the antibody clone C8/144B (Agilent, 1:200, 32 minutes at 37°C). CD8 was detected using anti-mouse HQ (Ventana Medical Systems) for 12 minutes at 37°C and subsequent anti-HQ horseradish peroxidase (Ventana Medical Systems) for 12 minutes at 37°C, followed by the Discovery Purple Detection Kit (Ventana Medical Systems). Slides were counterstained with Hematoxylin and Bluing Reagent (Ventana Medical Systems). All immunohistochemistry slides were uploaded to SlideScore for visual exploration.

### 4.3 TLS Clustering Approach

We employed an unsupervised learning strategy to identify TLS clusters with distinct immune cell composition. A k-Means algorithm was trained with the cellular densities (cells/mm2) of B-cells, CD4 T-cells, CD8 T-cells, FoxP3 T-cells, and macrophages in TLS using input from all TLS identified in the untreated cohort (n=754, **Figure 1A**, **Table 1**). Cellular densities per TLS (with a pseudo-count of 0.01 cells/mm2 to account for null densities) were transformed to a logarithmic scale and scaled by the standard deviation after subtracting the mean. The k-means clustering algorithm was trained by testing 1 to 10 centroids with a maximum of 300 iterations. An optimal number of k=5 clusters was selected based on a reduction or decrease of the total within-cluster sum of squares observed from k=5 to k=6 (**Supplementary Figure 6**), by visual exploration of the separation on a tSNE plot (**Figure 4D**), and by taking into account that only 5 features (distinct immune cell densities) were used to train the k-means algorithm.

To assign clusters to TLS identified in the treated NABUCCO cohort, cellular densities (with a pseudo-count of 0.01 cells/mm2 to account for null densities) were transformed to a logarithmic scale, followed by subtraction of means computed on the untreated, and scaling by the standard deviations computed on the untreated cohort. Then, we computed the distances between each TLS and each of the 5 centroids trained with the k-means clustering on the untreated cohort and predicted each TLS subtype by selecting the nearest centroid.

## Data Availability Statement

Multiplex Immunofluorescence raw data will be made available upon reasonable request for academic use by the corresponding author within the restrictions of the informed consent. A data access agreement will need to be signed with the Netherlands Cancer Institute, and reviewed by the institutional review board of the Netherlands Cancer Institute after approval. 

## Ethics Statement

The studies involving human participants were reviewed and approved by Institutional review board of The Netherlands Cancer Institute - Antoni van Leeuwenhoek. The patients/participants provided their written informed consent to participate in this study.

## Author Contributions

ND, MH, SE, LJ, BR, and CV: Interpreted and addressed clinical data. ND, SE, BR, and CV: Collected clinical data. AG-J, DV, LW, and KS: Performed and interpreted bioinformatics and statistical analysis. ND, AG-J, KS, MB, and MH: Analysed translational data. DP: Carried out immunohistochemical and multiplex immunofluorescence staining. ND, YL: Performed HALO AI Image analysis. KS and MB: Contributed to work related to Tertiary Lymphoid Structures. EH, AB, and MM: Supervised immunohistochemical/multiplex immunofluorescence quantifications, and assessed tissue availability. MM and JS: Carried out histopathological assessment and assessed tissue availability. KS and MB: Performed immunohistochemical/multiplex immunofluorescence work on the TLS panel. KS, MB, LW, LJ, and BR: Scientific input and critical review. MH, ND, and AG-J: Wrote the manuscript together with all co-authors, who approved the data accuracy, edited and approved the manuscript. All authors contributed to the article and approved the submitted version.

## Funding

MB was financially supported by the Swiss National Science Foundation (CRSII5_177208 and 310030_175565), the University of Zurich Research Priority Program ‘Translational Cancer Research’, the Cancer Research Center Zurich and Worldwide Cancer Research (18−0629). The NABUCCO study was financially supported by Brystol-Myers Squibb (BMS). The funder was not involved in the study design, collection, analysis, interpretation of data, the writing of this article or the decision to submit it for publication.

## Conflict of Interest

MH received research funding from Bristol Myers Squibb, AstraZeneca, 4SC and Roche, and consultancy fees from Bristol Myers Squibb, Roche, Merck Sharp & Dohme, Merck, AstraZeneca, Pfizer, Janssen and Seattle Genetics which were all paid to the Netherlands Cancer Institute. LW received research funding from Genmab.

The remaining authors declare that the research was conducted in the absence of any commercial or financial relationships that could be construed as a potential conflict of interest.

## Publisher’s Note

All claims expressed in this article are solely those of the authors and do not necessarily represent those of their affiliated organizations, or those of the publisher, the editors and the reviewers. Any product that may be evaluated in this article, or claim that may be made by its manufacturer, is not guaranteed or endorsed by the publisher.
