# Supplementary material for: The Tumor Immune Landscape and Architecture of Tertiary Lymphoid Structures in Urothelial Cancer
Source: Front Immunol. 2021 Dec 20;12:793964. doi: 10.3389/fimmu.2021.793964 (PMC8721669; doi:10.3389/fimmu.2021.793964)
Supplement: Supplementary file 1 [file DataSheet_1.docx]

Supplementary Material

# Supplementary Methods

## Image analysis of tumor and TLS regions upon multiplex immunofluorescence

Following multiplex immunofluorescent staining and VECTRA image acquisition (Akoya Biosciences, v3.0), image analysis was done in HALO (Indica Labs, v2.3). Using HALO, the tumor bulk was identified and the outermost edges were annotated manually. The tumor margin was defined as the region centered on the outermost border separating healthy tissue from malignant carcinoma. In HALO, the tumor margin was automatically computed from the outermost tumor edge, extending 125µm inside and beyond, covering 250µm centered on the border. Immune cell densities in tumor margins were assessed based on the tumor margin area, irrespective of tissue classifier. Based on observations, the tumor margin was arbitrarily selected to cover mostly immune cells just outside the tumor edge, without incorporating too much stroma that may dilute immune cell densities. The central tumor was defined as the area included within the outermost edges of tumor. Immune cells were quantified in central tumors for tumor and stroma, classified by the HALO random forest algorithm. automated tissue segmentation. This classifier was employed to discriminate stroma from Pan-Cytokeratin positive tumor regions. Square grids were computed in central tumors for spatial sampling to assess heterogeneity of immune subsets. In total, 30 square grid tiles were computed and normalized to fit the area within central tumor. Annotation of dense B-cell aggregates associated with tertiary lymphoid structures (TLS) formation was done manually, annotating the outer edge of dense B/T-cell presence that delineate TLS. For superficial TLS analysis, submucosal TLS were separated from TLS beyond the submucosa. The cellular density TLS immune subsets were analyzed based on the total number of cells in the total TLS area, by definition involving stroma. Tissue annotation and cell identification was performed together with a pathologist.

## Quantification of immune phenotypes

Immune phenotypes in our urothelial cancer cohorts were assessed based on multiplex immunofluorescence analysis, resulting in 1) Immune-excluded (≥5 times higher CD8 in CT-stroma or in tumor margin compared to CT-tumor), 2) immune-desert (Below median CD8 79 T-cells per mm2] in CT-tumor, not excluded), or 3) Immune-inflamed (Above median CD8 in CT-tumor, not excluded). The median cut-off for central tumor CD8 T-cells that separated inflamed from desert tumors was supported by visual inspection, as notable CD8 infiltration was not found in tumors assigned as immune-desert. Tumor immune phenotype assignment was conducted by an experienced uro-pathologist.

## Analysis of CD8/P1 co-expression

## Using HALO AI v3.2, we assessed CD8/PD1 positivity to assess CD8+PD1+ T-cell density in untreated and immunotherapy treated tumors. We annotated the central tumor based on the outermost border separating healthy tissue from invading tumor, as described in supplementary methods 1.1. For tissue segmentation, we annotated “tissue” and “tumor nests” within tissue to separate tissue and tumor form background on whole-slide sections. These annotations were used as a learning dataset to train a neural-network (Mininet) that is fast to run to identify tumor and stroma within central tumors. The distinct tissue classes were visualized as mark-up annotations and evaluated by a pathologist to facilitate an active learning cycle to optimize annotation accuracy. These mark-up annotations were then used as input to train a deep neural network (DenseNet), allowing more robust classifiers at higher resolutions. Cellular phenotypes were classified and analyzed using the HALO multiplex algorithm (v3.0.3). Segmentation of immune cells was done based on input from cellular parameters, including nuclei size, optical density and cellular roundness. CD8 and PD1 positivity in our cohorts was analyzed based on optimal marker color positivity thresholds established by a pathologist. The density of CD8+PD1+ T-cells was quantified in classified tissue regions separately.

## Quantification of central tumor and TLS on the untreated cohort

## The quantification of central tumor for one patient (ypT4N0) from the untreated cohort (Table 1, Fig 1A) was not carried out due to insufficient tumor abundance present in the tissue slide from the selected tissue block. Thus, central tumor quantifications were carried out on 31 patients from the untreated cohort (Table 1). Nevertheless, because notable TLS (n=22) could be quantified in the slide, TLS quantifications on TLS for the patient were included in the TLS analysis. Thus, TLS quantifications were carried out in 32 patients from the untreated cohort (Table 1).
