# Supplementary figures and images for: The Tumor Immune Landscape and Architecture of Tertiary Lymphoid Structures in Urothelial Cancer
Source: Front Immunol. 2021 Dec 20;12:793964. doi: 10.3389/fimmu.2021.793964 (PMC8721669; doi:10.3389/fimmu.2021.793964)

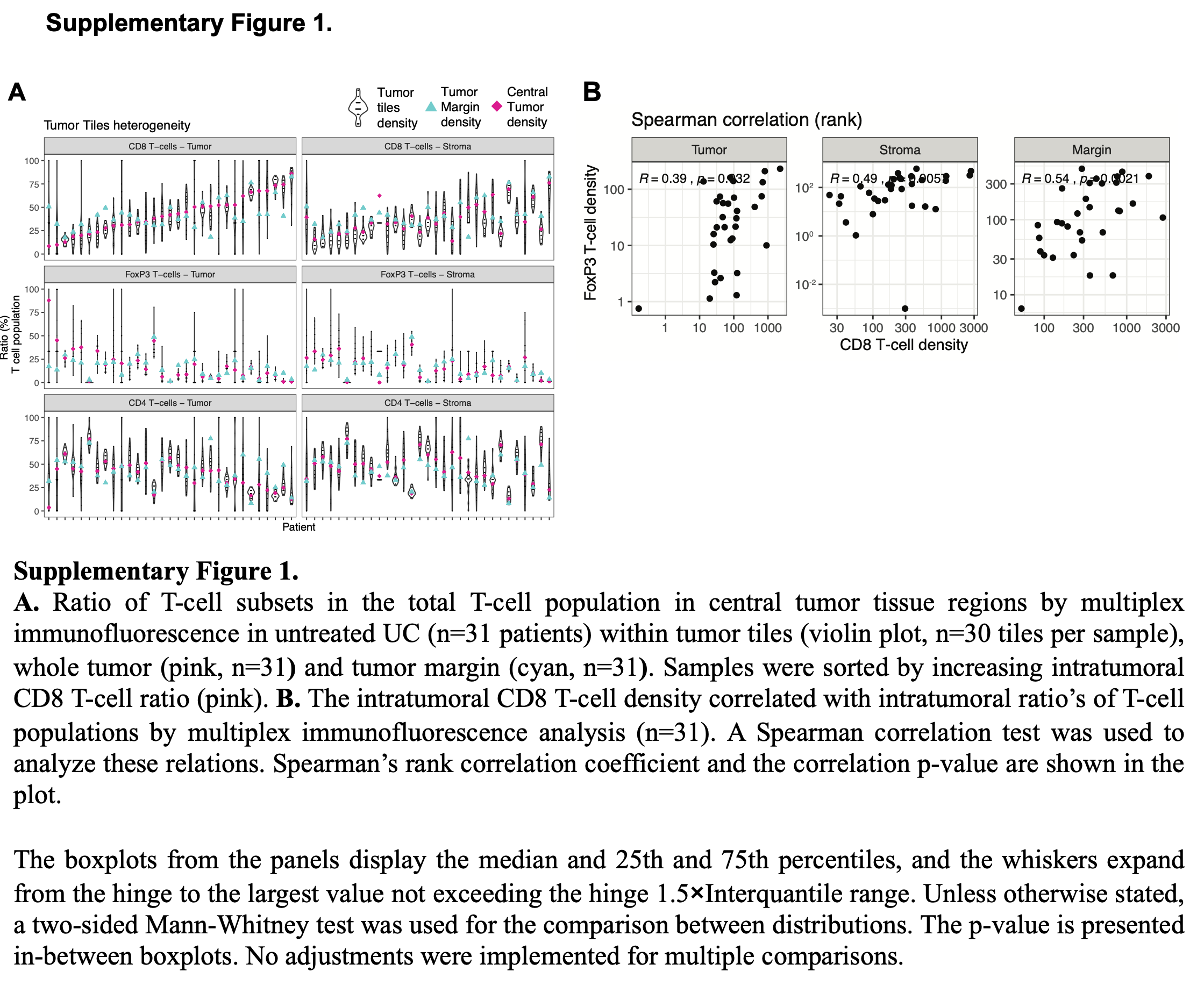

Supplement: Supplementary file 2 [file Image_1.tiff]

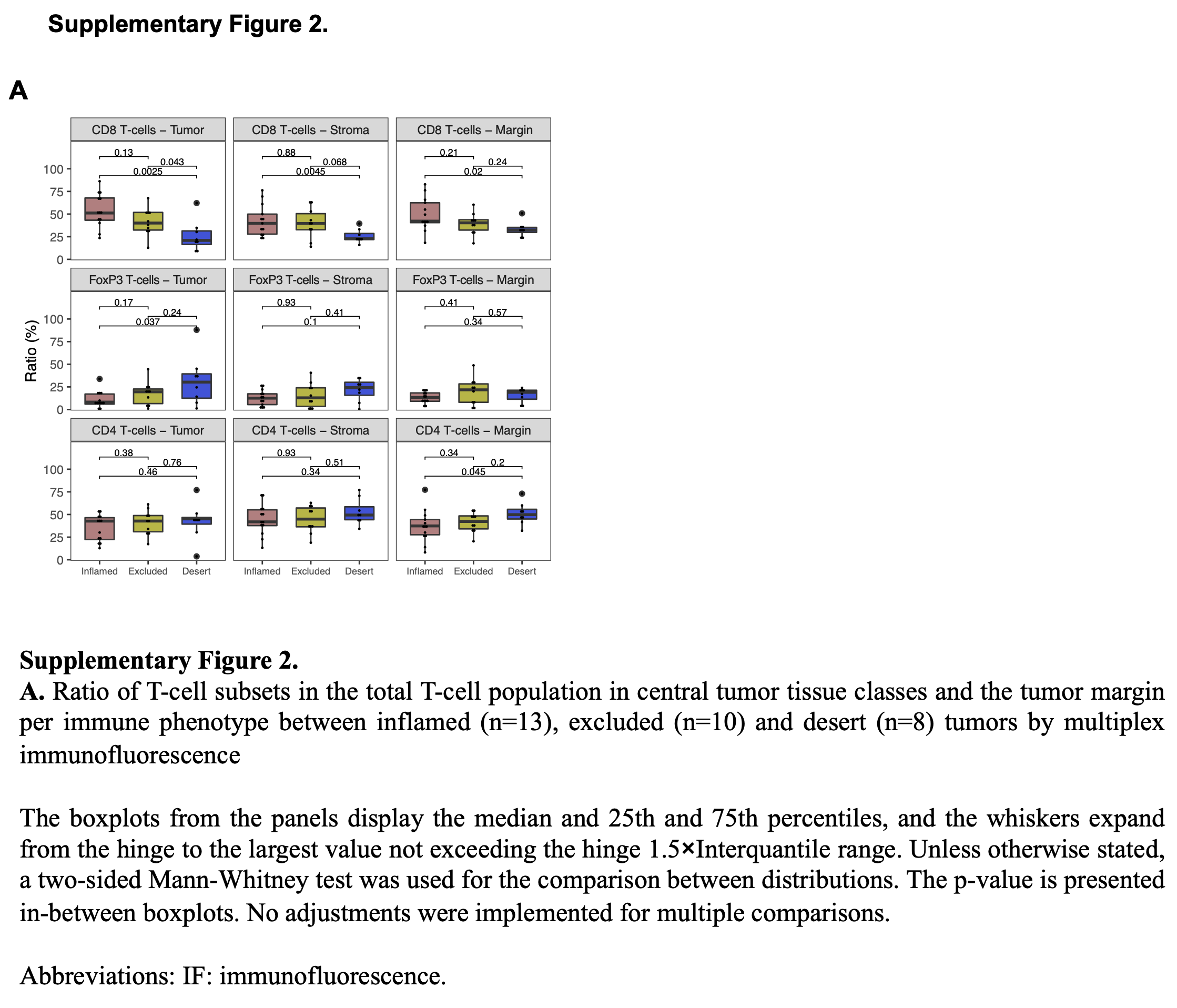

Supplement: Supplementary file 3 [file Image_2.tiff]

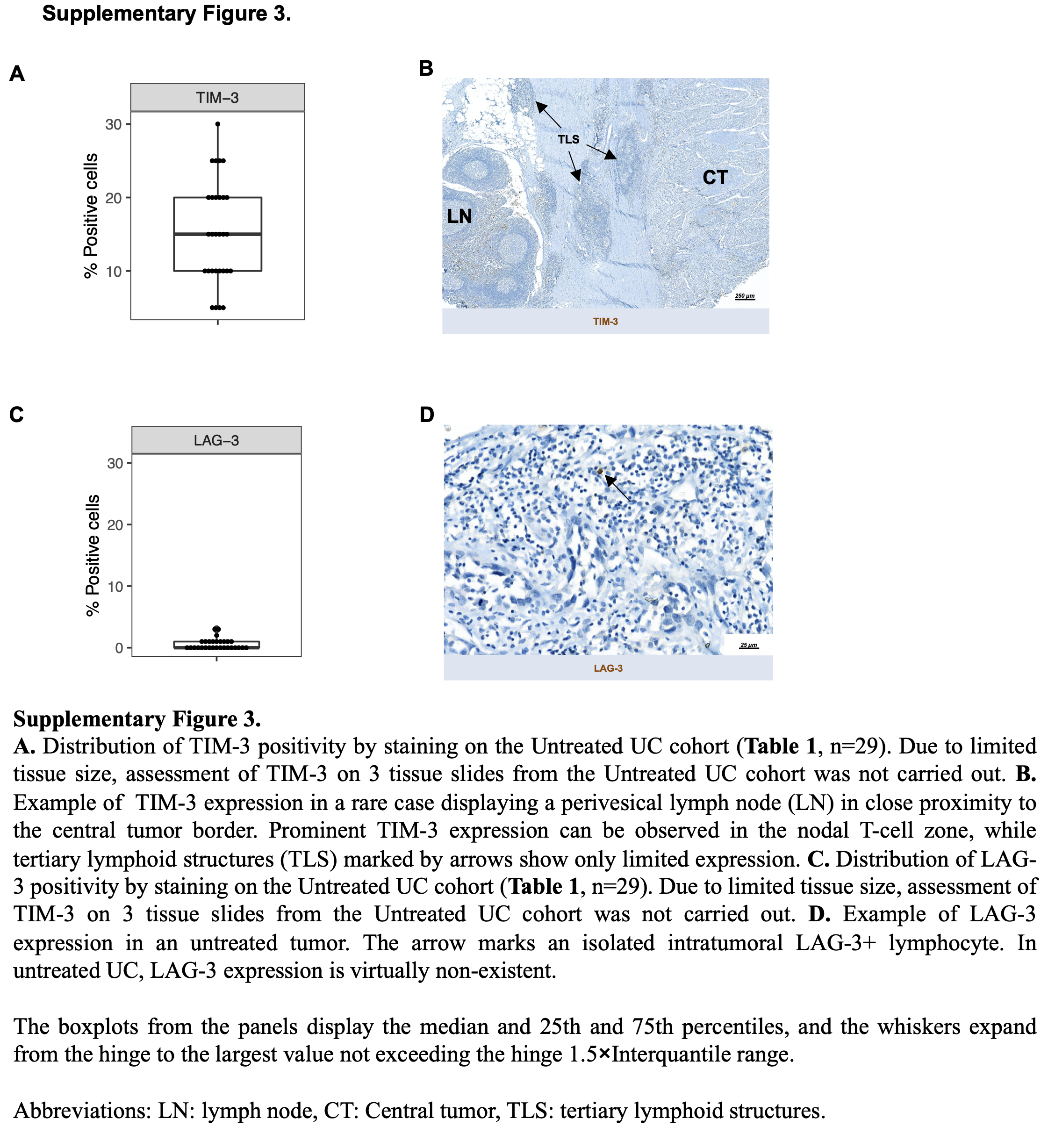

Supplement: Supplementary file 4 [file Image_3.tiff]

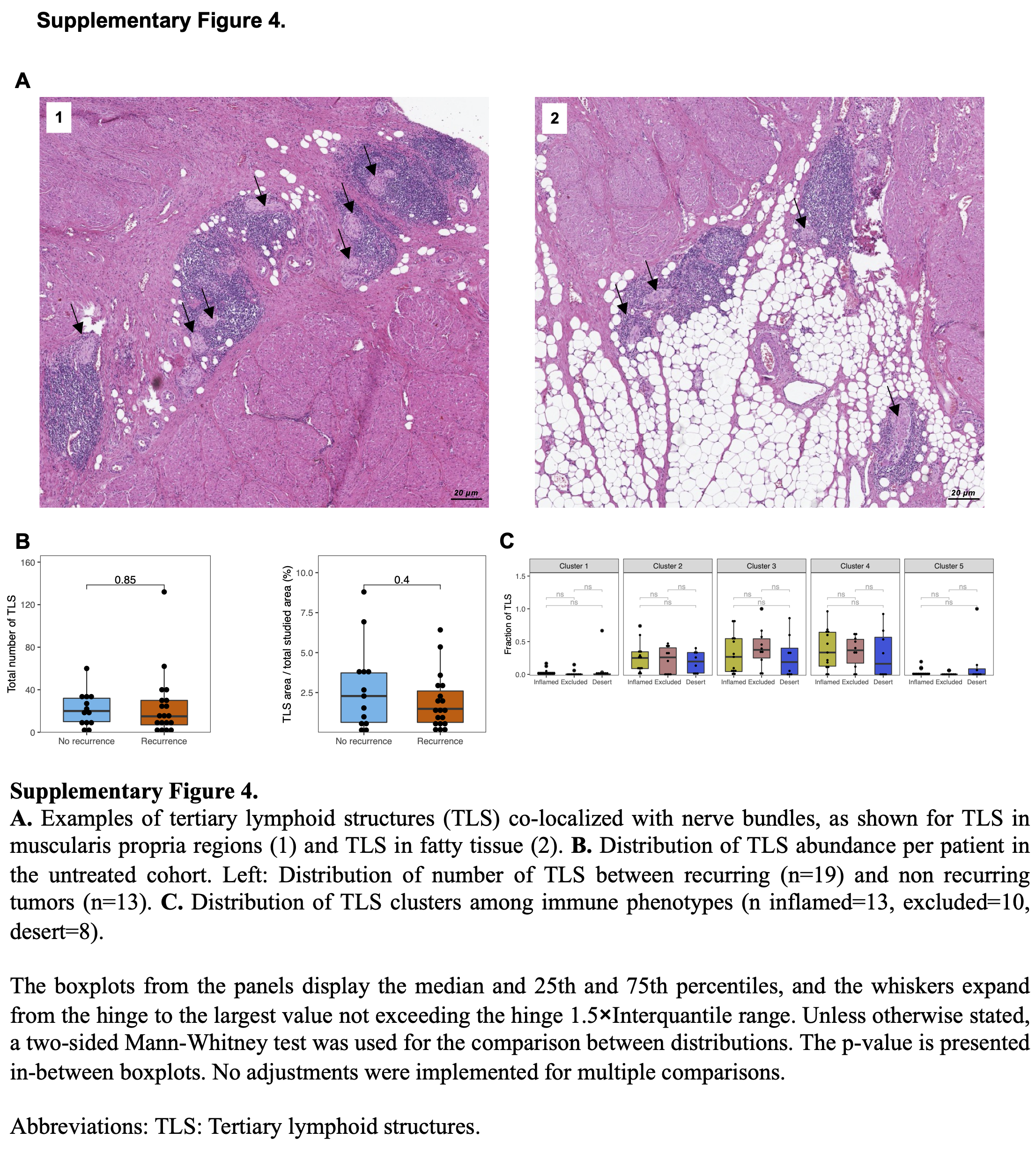

Supplement: Supplementary file 5 [file Image_4.tiff]

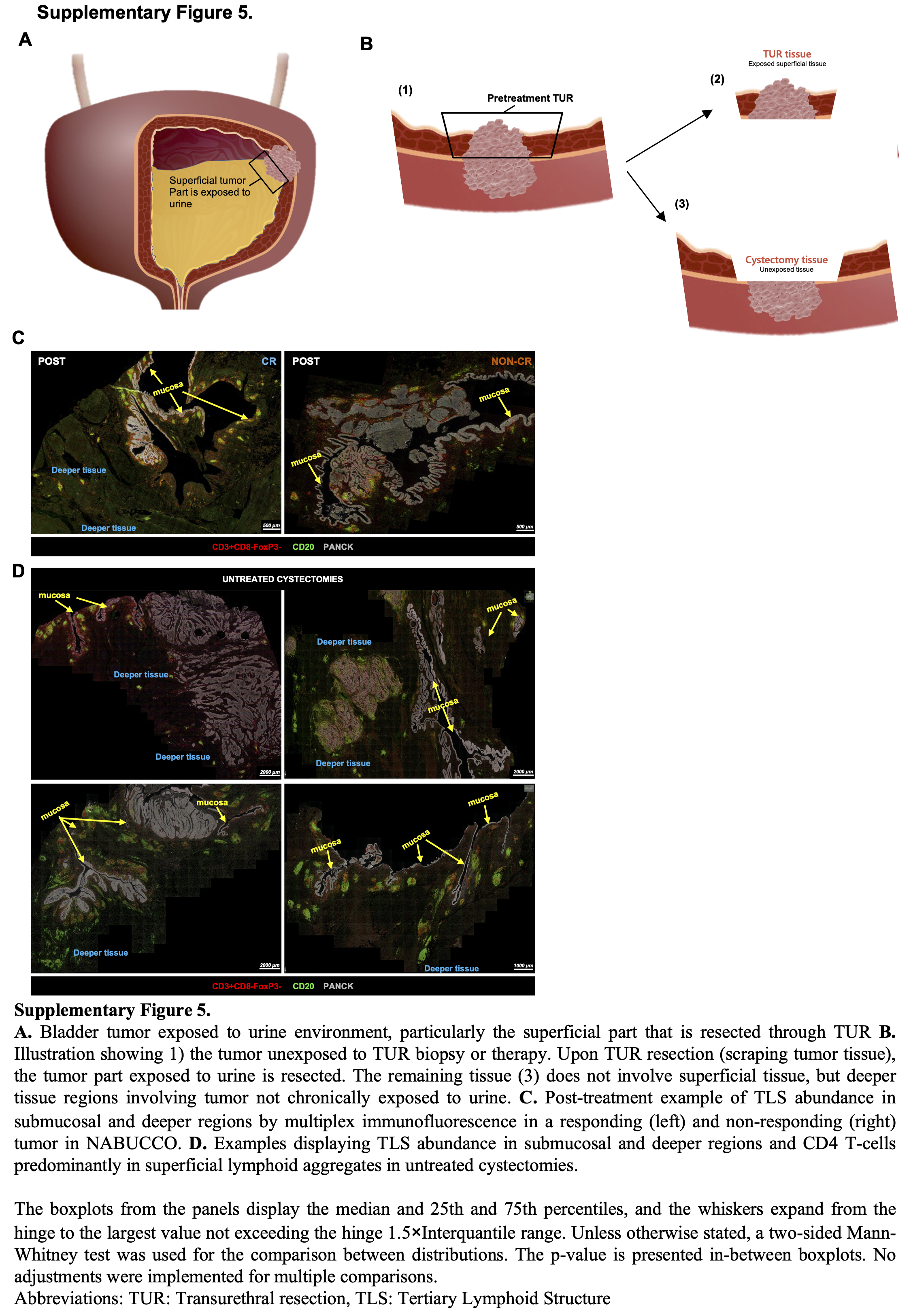

Supplement: Supplementary file 6 [file Image_5.tiff]

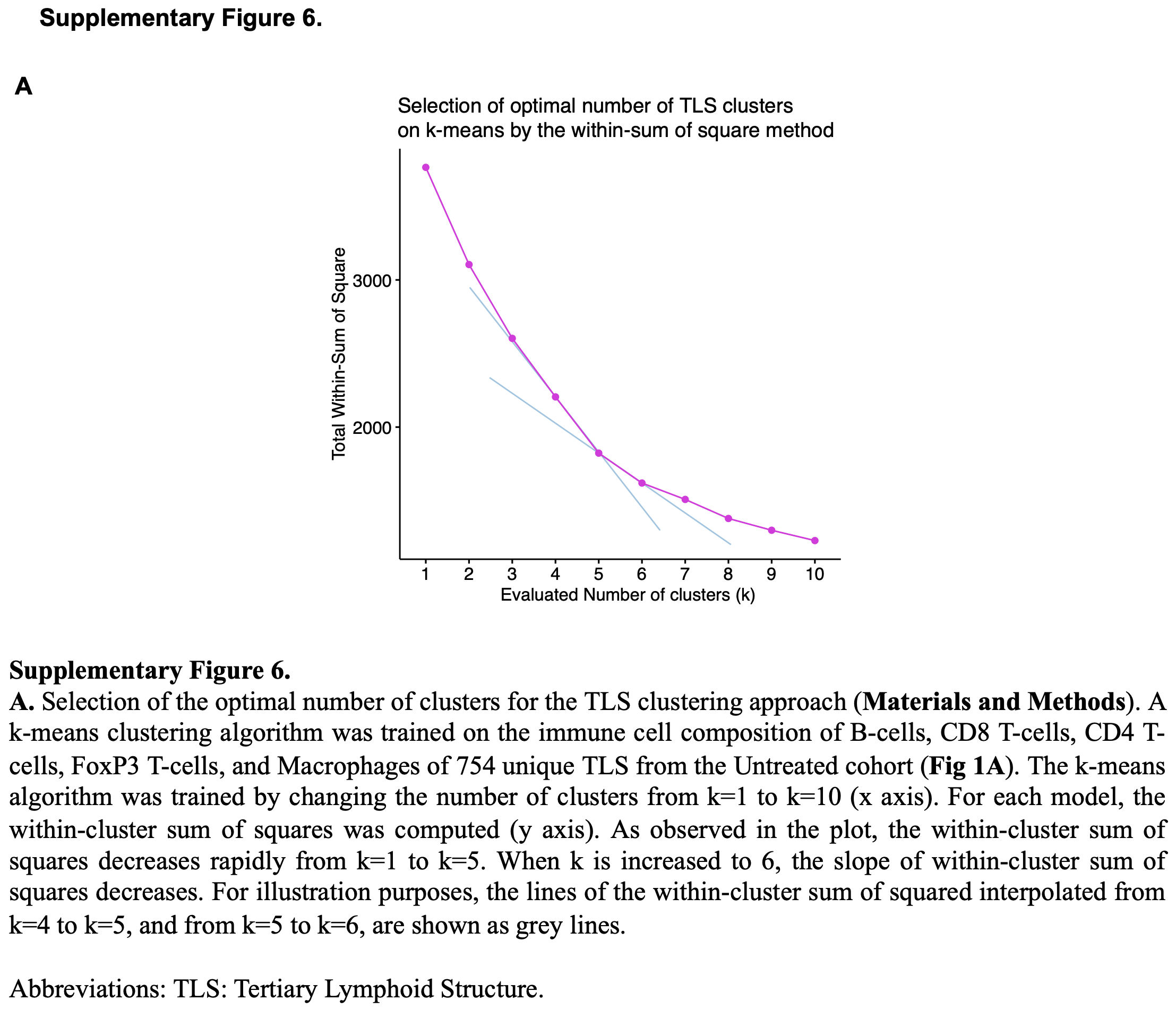

Supplement: Supplementary file 7 [file Image_6.tiff]
