# Supplementary material for: The Tumor Immune Landscape and Architecture of Tertiary Lymphoid Structures in Urothelial Cancer
Source: Front Immunol. 2021 Dec 20;12:793964. doi: 10.3389/fimmu.2021.793964 (PMC8721669; doi:10.3389/fimmu.2021.793964)
Supplement: Supplementary file 8 [file Table_1.docx]

Supplementary Material

## Supplementary Tables

### 3.1 Supplementary Table 1: Characteristics of an additional cohort of untreated tumors used for assessment of TLS maturation

| **Baseline characteristics** | **Total (n=40)** |
| --- | --- |
| Male sex, n (%) | 30 (75%) |
| Median age – years [range] | 62.50 [39-82] |
| Pathological T stage, (%) |  |
| pT1-4N0M0 | 25 (63%) |
| pT2-4N1-2M0 | 15 (38%) |
| Histology |  |
| Urothelial Carcinoma (%) | 40 (100%) |
| Adjuvant treatment |  |
| No adjuvant treatment | 34 (85%) |
| Adjuvant chemotherapy | 6 (15%) |
| Adjuvant treatment |  |
| No adjuvant treatment | 31 (78%) |
| Adjuvant chemotherapy | 5 (12%) |
| Adjuvant radiotherapy | 3 (7%) |
| Adjuvant chemotherapy and radiotherapy (%) | 1 (3%) |
